# Cluster of Acute Appendicitis Among High School Tibetan Students in Nanchang, China: Investigation, Control, and Prevention

**DOI:** 10.3389/fpubh.2022.889793

**Published:** 2022-04-15

**Authors:** Yitian Guo, Deqiang Ye, Guifang Yang, Guozhen Liu, Xiaochen Cui, Shiyun Tan, Yi Guo

**Affiliations:** ^1^Department of Gastroenterology, Renmin Hospital of Wuhan University, Wuhan, China; ^2^Department of Surgery, Infectious Disease Hospital Affiliated to Nanchang University (Nanchang Ninth Hospital), Nanchang, China; ^3^Department of Pathology, Zhongnan Hospital of Wuhan University, Wuhan, China; ^4^Swedish Bellevue Primary Care Clinic, Bellevue, WA, United States; ^5^Department of Epidemiology and Biostatistics, Wuhan University School of Public Health, Wuhan, China

**Keywords:** appendicitis, cluster, control, prevention, surveillance

## Abstract

**Objective:**

Infectious etiology of acute appendicitis is a current hot topic. The most of study on appendicitis came from sporadic patients and focused on clinical treatment rather than control and prevention of appendicitis in the population. The present study aims to investigate the epidemiological features of cluster of acute appendicitis, risk factors, and evaluate effectiveness of control and prevention in population.

**Methods:**

We conducted longitudinal study on a cluster of acute appendicitis among Tibetan students at a high school in eastern China, which was divided into three stages: 1. We retrospectively collected epidemiological data and clinical data to explore risk factor and possible transmission route in August of 2005; 2. We conducted targeted measures from August of 2005 and analyzed incidence trend from 2000 to 2010; 3. Since no new patients occurred in 2011, we conducted surveillance from the beginning of 2012 until July 2018.

**Results:**

Among 973 Tibetan students, there were 120 patients with more female patients (102 of 499, 20.4%) than male patients (18 of 474, 3.8%) from January of 2000 to December of 2010. The 4-year cumulative incidence rates in female students enrolled in 2001, 2002, 2003, 2004, 2005, 2006 were 26.8% (11 of 41), 27.1% (13 of 48), 44.7% (21 of 47), 42.4% (14 of 33), 23.1% (9 of 39), and 19.3% (11 of 57), respectively before their graduation. There was a clustering feature. Mutual contact with patients before the onset of symptoms was an important risk factor (Adjusted OR 4.89, 95% CI: 1.67–14.35). Transmission route may be fecal-oral infection. Before conducting targeted measures, the incidence rate increased from 2000 and peaked in 2005. After conducting targeted measures, the incidence rate decreased year by year until 2010. Under surveillance from January of 2012 to July of 2018, only four sporadic patients occurred at this school.

**Conclusion:**

This cluster of acute appendicitis had features of an infectious disease in epidemiology, which can be controlled and prevented by targeted measures. Our study may also be used for prevention of sporadic patients and be generalized in general population as cluster of appendicitis occurred in many provinces of China.

## Introduction

Acute appendicitis is the most common surgical abdominal emergency worldwide (1). Little is known about the exact cause of acute appendicitis, therefore control and prevention measures for acute appendicitis in population are difficult to be taken. In addition to an obstructive process (2), other possible risk factors for appendicitis include high sugar intake and low dietary fiber (3), viral (4–7), bacterial (8–10), and parasite infestations (11). In recent years, bacterial infection has also been proposed as the primary cause of acute appendicitis (12, 13). A few studies showed a significant change of the intraluminal bacterial composition in inflamed appendices (14–16). Oral microbes, including Fusobacterium, Gemella, Porphyromonas, and Peptostreptococcus were significantly increased in patients with acute appendicitis (14–20). A possible route of infection of acute appendicitis is a viable migration of oral pathogens from the oral cavity through the stomach postprandially (20). If appendicitis has an infectious etiology, both sporadic and clustered patients should appear.

In 1984, a cluster of true appendicitis occurred in a town of 8,000 people in the United States (21). Thirteen patients with acute appendicitis occurred during a 3-month period, which was associated with food intake. Regarding this clustering of acute appendicitis, the Centers for Disease Control and Prevention (CDC) published editorial note that the cluster offered a unique opportunity to identify possible risk factors and to search for precipitating infectious agents, and encouraged reporting such cluster to CDC (22). In 1997, we found a more severe cluster of acute appendicitis at a high school in the city of Wuhan, central China in which 10 patients occurred among 290 students from April 10, 1997 to June 11, 1997. We followed up to June, 2000, 20 sporadic patients occurred (23). As the cluster of acute appendicitis ended before we began investigation, so targeted control and prevention studies could not be conducted. In 2012, Fusobacteria were found in appendices of these clustered patients (13), which suggested that the cluster of acute appendicitis may be caused by an infectious agent.

Considering that the patients in two clusters in the United States and China occurred at schools, we focused on looking for new clusters of acute appendicitis at schools from the beginning of 2005. We learned that Tibetan students were enrolled at the schools of 20 provinces and municipalities of China since 1985 and incidence rates of acute appendicitis increased at many of these schools. We contacted four schools in four provinces where cluster of acute appendicitis occurred there for years. Finally, we selected a high school in the city of Nanchang, Jiangxi Province, eastern China, as this high school and the designated hospital that treated student with acute appendicitis were willing to collaborate with this investigation.

To date, the vast majority of studies on appendicitis have come from sporadic cases and have focused on clinical treatment rather than control and prevention of appendicitis in the population. The first aim of this study was to describe the epidemiological features of the cluster of acute appendicitis, and to identify the associated risk factors and the possible transmission route. The second aim was to conduct targeted measures to demonstrate effectiveness of control and prevention for acute appendicitis in certain population. The third aim was to find common settings of cluster of acute appendicitis in order to study on control and prevention of acute appendicitis in other populations.

## Materials and Methods

### Study Background and Design

The school enrolled Tibetan students from 1985. Length of schooling was 4 years. Most of them came from Tibetan countryside. They boarded in the school. The living condition and the hygiene were good overall. The student building was partitioned by a wall on each floor with one side for female students and another side for male students. There were six students in each room with wash stand, toilet and bathroom. The management of building is very strict. The male students and the female students are not allowed to enter rooms of the other side without permission. Each Tibetan student ate same set of food for each meal at the same time in the same dining hall. The school arranged life guardians for Tibetan students. Life guardians were responsible for supervising and guiding students' diet hygiene, dormitory hygiene and personal hygiene, correcting and guiding students' behavior, life style, enforcing rules and regulations after school hours, writing students' activity summary and weekly report to the lead teacher.

We conducted longitudinal study design to investigate epidemiological features, risk factors and effectiveness of intervention for the cluster of appendicitis, which was divided into three stages: 1. We began this study in August of 2005. Retrospectively, we collected epidemiological data and clinical data prior to August of 2005 in order to explore risk factor; 2. After finding risk factor, we conducted intervention to control the cluster of appendicitis in August of 2005; 3. Since the cluster of acute appendicitis was under control in 2011, we conducted surveillance for acute appendicitis from the beginning of 2012 until July 2018 to monitor whether intervention measures could prevent re-occurrence of cluster of appendicitis. Our study was conducted according to epidemiological steps of a cluster investigation and reported compliant with STROBE (24–26).

### Epidemiological, Clinical, Laboratory, and Pathological Investigation

The epidemiological data included person and time distributions of patients, exposure history and environmental sanitation at this school, which were obtained by visiting the school and interviewing the patients and Tibetan students. Patients were identified by reviewing hospital records. The clinical data included patient history, physical examination, laboratory tests, pathology examination, which were collected on a data collection form. Routine blood tests were performed before surgery for all the patients.

Patients were defined as those who had typical clinical manifestations and who had surgery (appendectomy) and pathological features of acute appendicitis. All pathological slides from appendectomy were re-examined in Department of Pathology, Zhongnan Hospital of Wuhan University. The study was approved by ethic boards at the designated hospital and Wuhan University School of Medicine. Written informed consent for investigating risk factors was obtained from guardians of each respondent.

### Investigation of Causes of Cluster in Tibetan Students

In August 2005, 72 students were in school for the summer vacation. We interviewed the students and their life guardians to investigate students' lifestyle and health habit and explore possible causes of the cluster, especially causes of higher incidence in female students. Among them, there were 28 patients who had acute appendicitis. Most students and their life guardians thought that the possible causes of higher incidence in female students were as follows: (1) Female students had a habits of staying in bedrooms, in contrast to male students who like outdoor activity; (2) Female students had a habit of eating snacks and sharing them with each other whereas male students did not; (3) The patients liked staying in bedrooms and eating snacks and sharing them more than healthy students; (4) Most patients had a contact history with their preceding patients before onsets of symptoms; (5) Toilets were built in bedrooms and most students do not have a habit of washing hands before eating snack, before meals and after bowel movement.

According to their answers, we designed questionnaire to investigate the following exposure factors between patients and healthy students: gender, eating snack (one bag per day on average), engaging in outdoor activities (after school 5 days a week on average), contact history (contacting their preceding patients in bedroom or living with them in bedroom or escorting patients to the hospital), health habit (washing hands before eating snack, before meals, and after bowel movement).

### Intervention Measures

Based on the investigational results and our suggestion, the school started intervention measures including regular health education for all students since August of 2005, which was conducted by life guardians, especially when new students entered school every year or new patients occurred. The measures were as follows: 1. Treating potential source of infection: life guardians escort patient to the hospital soon to reduce contact opportunities with healthy students once patient appear. 2. Conducting health education to block possible routes of transmission and protect potential susceptible population: (1) washing hands before meal and after bowel movement; (2) flushing and cleaning toilets after using toilet; (3) avoiding contact with patients and cleaning fomites once patients appear; (4) washing hand and clothes if contacting patients. (5) Limiting eating snacks and increasing outdoor activities.

### Evaluation of Effectiveness of Intervention Measures

After intervention, we followed up Tibetan students three times a year, including one visit (in July each year) to school and two telephone calls (in March and November each year) in order to evaluate effectiveness of intervention. The following data were collected: incidence rate, date of onset of new patients, risk factors and intervention against risk factors and so forth. During period of surveillance between 2012 and 2018, we followed up in March and November each year by two telephone calls.

### Statistical Analysis

Statistically significant differences in incidence rates between the male and female students were assessed using chi-square tests. Univariate and multivariate logistic regressions were conducted to explore potential risk factors of acute appendicitis. Firth's logistic regression, using a penalized likelihood estimation method, was applied as an appropriate method to deal with small sample size. In multivariate logistic regression, all potential factors were included.

Interrupted time series analysis was applied to explore change in incidence rate before and after health education. An interaction between year and group (0-before health education, 1-after health education) was created for hypothesis test. Durbin-Watson statistics and residual autocorrelations were assessed and a generalized ordinary least square regression with Prais-Winsten estimation was applied to explore whether or not the interaction was statistically significant. A *P-*value <0.05 was considered statistically significant. SPSS 26.0 was used for statistical analysis.

## Results

### Epidemiological Findings

**Figure 1:**
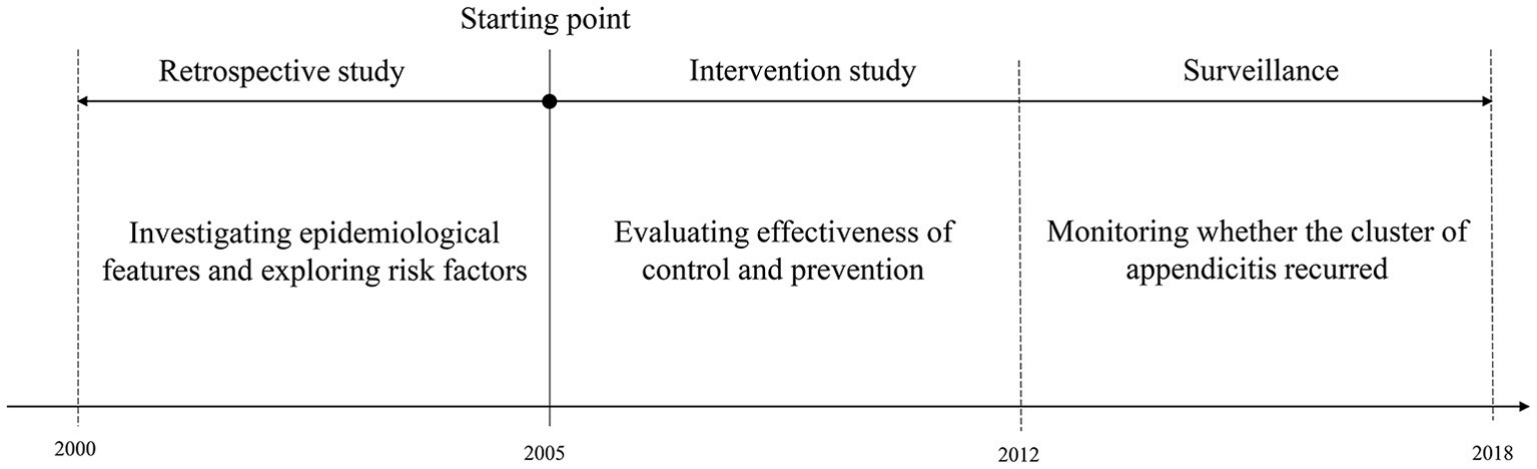
Flow chart of three stages of longitudinal study on the cluster of appendicitis.

According to study design, three stages of longitudinal study was showed in Figure 1. Epidemiological steps of the cluster investigation were listed in Table 1. Step 1 to step 8 in Table 1 match stage 1 in Figure 1. Step 9 and step 10 match stage 2 and stage 3, respectively.

**Table 1:** Epidemiological steps of a cluster investigation.

1. Prepare for field work
2. Establish the existence of a cluster
3. Verify the diagnosis
4. Construct a working case definition
5. Find cases systematically and record information
6. Perform descriptive epidemiology
7. Develop hypotheses
8. Evaluate hypotheses epidemiologically
9. Compare and reconcile with laboratory and/or environmental studies
10. Implement control and prevention measures 11. Initiate or maintain surveillance

Before 2000, there were clusters of tuberculosis, varicella, mumps and so forth. From 1998, the incidence rate of acute appendicitis increased in Tibetan students. After 2000, the patients were admitted to the designated hospital for treatment. From January of 2000 to December of 2010, 973 (474 male and 499 female) Tibetan students were enrolled at this high school. Among them, there were 120 students suffered from acute appendicitis (102 females and 18 males). Patients' age ranged from 12 to 17 years. The mean age (SD) was 14.3 (1.1) years. Person and time distribution of the patients were showed in Table 2. There was a female preponderance (female 20.4%, 102 of 499; male 3.8%, 18 of 474; chi-square = 62.280, *P* ≤ 0.001). The incidence peak appeared in 2005. In 2003, epidemic of severe acute respiratory syndrome (SARS) occurred in China. Because refusal to visitors, disinfection and personal protection were carried out at this school for prevention of epidemic of SARS, there was only one patient with acute appendicitis. The 4-year cumulative incidence rates in female students enrolled in 2001, 2002, 2003, 2004, 2005, 2006 were 26.8% (11 of 41), 27.1% (13 of 48), 44.7% (21 of 47), 42.4% (14 of 33), 23.1% (9 of 39), and 19.3% (11 of 57), respectively before their graduation. There were no obvious seasonal variations of patient occurrence among the Tibetan students.

**Table 2:** Person and time distribution of the patients in Tibet students.

**Year**	**Male**		**Female**		**Total**	
	**Number of students**	**Number of patients (%)**	**Number of students**	**Number of patients (%)**	**Number of students**	**Number of patients (%)**
2000	136	1 (0.7)	158	8 (5.1)	294	9 (3.1)
2001	147	2 (1.4)	158	9 (5.7)	305	11 (3.6)
2002	153	0 (0.0)	163	14 (8.6)	316	14 (4.4)
2003	158	0 (0.0)	173	1 (0.6)	331	1 (0.3)
2004	165	1 (0.6)	173	18 (10.4)	338	19 (5.6)
2005	176	3 (1.7)	168	19 (11.3)	344	22 (6.4)
2006	189	4 (2.1)	171	13 (7.6)	360	17 (4.7)
2007	192	3 (1.6)	184	10 (5.4)	376	13 (3.5)
2008	183	1 (0.6)	201	3 (1.5)	384	4 (1.0)
2009	175	2 (1.1)	207	6 (2.9)	382	8 (2.1)
2010	152	1 (0.7)	201	1 (0.5)	353	2 (0.6)

A history of mutual contact was found in these patients and the onset time of them is close. Most of the patients were classmates (Table 3) or roommates (Table 4). Because there were large number of patients occurring from 2000 to 2010, we only chose the patients occurring in 2004 to be presented in Table 3. With the exception of case 17, all patients occurred in cluster. In Table 4, contact history recall is reliable as 17 were roommates among 19 patients who had contact history.

**Table 3:** Dates of onset of the patients in Tibet students in 2004.

**Case no**.	Grade^*^	**Class**	**Dates of onset**
1	2003	1	04-05-2004
2	2003	1	04-07-2004
3	2002	2	05-09-2004
4	2002	2	05-09-2004
5	2002	2	06-14-2004
6	2002	2	06-19-2004
7	2003	2	06-28-2004
8	2003	1	06-29-2004
9	2003	1	07-02-2004
10	2002	1	07-03-2004
11	2003	2	07-05-2004
12	2002	1	07-20-2004
13	2002	1	07-22-2004
14	2002	1	07-27-2004
15	2003	2	08-13-2004
16	2003	1	08-16-2004
17	2003	2	09-18-2004
18	2003	1	12-04-2004
19	2004	2	12-17-2004

* *Grade is classified by the years when students were enrolled. Each grade is further grouped to different classes*.

**Table 4:** Exposure among acute appendicitis patients and controls.

	**Patients (*n* = 28)**	**Controls (*n* = 44)**	**OR (95% CI)**	***P***	**Adjusted OR (95% CI)**	***P***
**Gender** ^†^						
Female	27 (96.4%)	36 (81.8%)	4.27 (0.64, 28.45)	0.13	1.04 (0.08, 13.78)	0.98
Male	1 (3.6%)	8 (18.2%)	Reference		Reference	
**Eating snack**						
Yes	20 (71.4%)	30 (68.2%)	1.15 (0.41, 3.22)	0.80	1.19 (0.37, 3.82)	0.77
No	8 (28.6%)	14 (31.8%)	Reference		Reference	
**Outdoor activity**						
Yes	26 (92.8%)	34 (77.3%)	3.22 (0.70, 14.80)	0.13	2.59 (0.32, 20.82)	0.37
No	2 (7.2%)	10 (22.7%)	Reference		Reference	
Contact history^*^						
Yes	19 (67.9%)	12 (27.3%)	5.34 (1.91, 14.91)	0.001	4.89 (1.67, 14.35)	0.004
No	9 (32.1%)	32 (72.7%)	Reference		Reference	
**Health habit**						
Yes	25 (89.3%)	40 (90.9%)	0.81 (0.17, 3.91)	0.79	0.83 (0.15, 4.68)	0.84
No	3 (10.7%)	4 (9.1%)	Reference		Reference	

† *Gender, eating snack, outdoor activity, and health habit were adjusted for multivariate analysis*.

* *In patient group, 17 were roommates among 19 patients who had contact history*.

### Clinical, Laboratory, and Pathological Features of the Clustered Patients

The patients' clinical signs and symptoms had features of typical acute appendicitis, whereas axilla temperature, white blood cell counts and neutrophil level were normal among the most patients. Pathologically, the resected appendices exhibited focal or diffuse hemorrhages in mucosa and/or submucosa, and infiltration by eosinophil and by lymphocytes. These features were identical to the features of the clustered patients in Wuhan (23). Among these patients, 1.7% (2 of 120) had acute gangrenous appendicitis, 6.7% (8 of 120) had acute suppurative appendicitis and the rest had acute simple appendicitis.

### Analysis of Risk Factors of the Cluster

The investigational result of risk factors for 72 students was shown in Table 4. In univariate analyses, contact history was the only risk factor of acute appendicitis (OR, 5.34; 95% CI: 1.91–14.91; *P* = 0.001). Gender, eating snack, outdoor activity, and health habit were not associated significantly with risk of acute appendicitis. In multivariate analyses, contact history was also the only risk factor of acute appendicitis (OR, 4.89; 95% CI: 1.67–14.35; *P* = 0.004).

### Effectiveness of Intervention Measures

The reason why eating snack, outdoor activity and health habit were not associated with acute appendicitis is likely due to the fact that the vast majority of patients and healthy students had these factors. Therefore, statistical differences in these factors between patients and healthy students cannot be found. However, once a new student came along, these factors may increase the risk of acute appendicitis. So we also took targeted measures to these factors.

**Figure 2:**
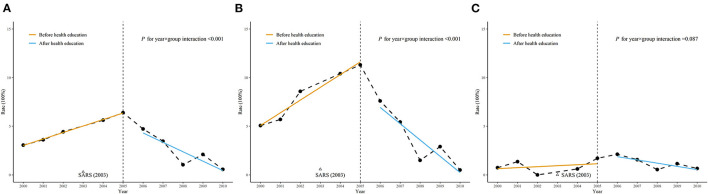
Comparison of the trends of incidence rate before and after health education. **(A)** Total participants; **(B)** Female participants; **(C)** Male participants.

Comparison of results before and after intervention was shown in Tables 2, 5 and Figure 2.

**Table 5:** Analysis of interaction between group and time among Tibetan students.

**Variable**	**Total**		**Female**		**Male**	
	**Coefficient (β)**	***P***	**Coefficient (β)**	***P***	**Coefficient (β)**	***P***
Group	−1.24	0.113	−3.49	0.02	1.15	0.167
Time	0.69	0.001	1.38	<0.001	0.09	0.535
Group*Time	−1.63	<0.001	−3.00	<0.001	−0.43	0.087

*Group: 0- before health education, 1-after health education*.

Using generalized ordinary least square regression with Prais-Winsten estimation, the interaction between group and time were significant among total students and female students and not significant among male students.

Before intervention, the incidence rate increased year by year from 2000 and peaked in 2005, with the exception in 2003 when there was a reduction of the incidence rate by the implementation of no visitor policy, disinfection and personal protection due to the SARS epidemic. After intervention from 2005, the incidence rate showed a decreasing trend year by year. No new patients occurred in 2011. Under surveillance from January of 2012 to July of 2018, only four sporadic patients occurred at this school.

## Discussion

We presented the epidemiological features, risk factor and effectiveness of control and prevention of the cluster of acute appendicitis among Tibetan students from January of 2000 to July of 2018. From 2000 to 2010, cluster of acute appendicitis continued for 11 years. The incidence rate (20.4%) among female students was significantly higher than that (3.8%) of male students. It was surprised that the 4-year cumulative incidence rates in female students enrolled in 2001, 2002, 2003, and 2004 were 26.8% (11 of 41), 27.1% (13 of 48), 44.7% (21 of 47), and 42.4% (14 of 33), respectively before their graduation. The incidence rates were much higher than that in the two clusters of acute appendicitis in the United States and Wuhan of China (22, 23). According to the literature we retrieved, this was most lasting cluster of acute appendicitis with the highest incidence.

The occurrence of clustering is usually features of infectious diseases. Our study showed clustering features of acute appendicitis. Many patients had a contact history with preceding patients. Univariate and multivariate analysis showed that the contact history was the only risk factor, suggesting that the cluster of acute appendicitis may result from infectious agent. Finding of Fusobacteria among clustered patients in Wuhan favors this hypothesis (13). No-visitors policy, disinfection, personal protection for prevention of SARS in 2003 and our intervention after 2005 can reduce incidence rate and ultimately control the cluster, further suggesting infectious etiology of the cluster.

The reason for the cluster of acute appendicitis among Tibetan students may be that they were not adapted to the new environment, which increased their susceptibility to potential infectious agents. The reason for higher incidence rate of female students is likely due to the following: Female students' lifestyle and personal health habit increased the chance of contact with patients and the risk of transmission, respectively. According to the school's management measures, male students and female students were not allowed to enter rooms of the other side without permission, the chance of contact between male students and female students was restricted. Female students had a habit of staying in bedrooms and mutual contact was more frequent than male students, therefore acute appendicitis occurred mainly among female students.

For infectious diseases, in the case that no pathogen is found, measures for control and prevention can be conducted by controlling source of infection, protecting susceptible population, and blocking transmission route. For the cluster of acute appendicitis, the patient was the potential source of infection. The healthy students, especially new students were the potential susceptible population. Regarding the transmission route, a common source of clustering caused by-water or food-borne transmissions can be excluded because female students and male students used one canteen and water source while female students had higher incidence rate than male students. In general, transmission route of gastrointestinal tract infectious diseases is often fecal-oral. This is also likely true for clustered appendicitis as microbes in gastrointestinal tract and in oral cavity were found in the inflamed appendices (14–20, 27–32). In addition, the cluster of appendicitis in the United States was associated with food intake. Therefore, it is possible the clustered appendicitis resulted from fecal-oral infection or migration of oral microbes through the stomach postprandially. After we took measures against the risk factor, potential source of infection, transmission route and susceptible population. The rising trend of appendicitis was curbed and incidence rate decreased year by year. The cluster was finally controlled and doesn't happen anymore. To our knowledge, this is the first report of appendicitis control and prevention in a certain population.

What need to be explained is: Why cluster of acute appendicitis is not so often as the other intestinal infectious diseases if acute appendicitis is caused by infectious agents? The possible reason is that the appendix is small, hidden, and pathogens are not easy to reach. There was evidence to support this hypothesis. Salmonella Typhi, Shigella dysenteriae, and so forth can cause clusters of typhoid fever and dysentery respectively, and also caused sporadic acute appendicitis (27–32). However, these bacteria never caused cluster of acute appendicitis. This finding can explain why cluster of acute appendicitis is not so often as the other intestinal infectious diseases.

Cluster of acute appendicitis occurred more frequently than we realized in this paper. In addition to clusters in Wuhan and Nanchang, other clusters of acute appendicitis occurred in five provinces and autonomous regions in China, which often occur in collective living units, such as schools and military camps (33–39). So many clusters of acute appendicitis and common settings shows that the study of risk factors and prevention and control of acute appendicitis in the population is sustainable. Among these clusters of acute appendicitis, some of them persisted for years and decades (34, 36–39). Two clusters were caused by eating overnight food (33). Most authors who reported the clusters of appendicitis were surgeons. They only discussed medical history and clinical features of the clustered patients, but did not discuss the epidemiological features and the associated risk factors in detail. Compared with our study, public health significance of the cluster of appendicitis was not fully paid attention to. Our study is expected to change readers' and clinicians' understanding of the cluster of appendicitis. For a disease, if there are clustered patients, there must be sporadic patients, so the measures for clustered patients may also be used for prevention of sporadic patients. Because acute appendicitis is not endemic disease, cluster of acute appendicitis can also occur worldwide. This suggests that our experience may be generalized in China and other countries.

### Strengths

- Due to its high incidence and long duration, the cluster provided ideal conditions for the study of risk factors and prevention and control of acute appendicitis in a certain population.
- The patients occurred in cluster. The contact history of the patients prior to the onset was reliable, since most patients had roommate or roommates with acute appendicitis, the order of the onset can be easily determined. In the investigation of risk factors, the OR value of contact history is 5.34 and the adjusted OR value is 4.89 respectively, therefore, the association between contact history and occurrence of acute appendicitis is significant.
- After intervention, the incidence rate of acute appendicitis continued to decline until the cluster was controlled, so the causal association between intervention and control of acute appendicitis was significant, which should not be caused by chance.

### Limitation

- Although occurrence of clustering often results from infectious diseases and many patients had a history of mutual contact before the onset of acute appendicitis, infectious etiology of the cluster of acute appendicitis was still a hypothesis as the associated pathogen was not isolated.
- As the investigation on risk factors of the cluster occurred in 2005 summer vacation and only 72 students were at school, so the sample size was not large, which might affect the power of test. For examples, although OR values of gender and outdoor activities were not small, there were no significant difference between patients and healthy students.

In summary, we investigated the cluster of acute appendicitis among Tibetan students at a high school in eastern China. The cluster lasted for 11 years. The incidence rate of acute appendicitis was much higher among female students. Female students' lifestyle and the school's management measures for Tibetan students resulted in higher incidence rate among female students. Contact history with patients was risk factor of appendicitis. The patients occurred in cluster. The possible transmission route may be fecal-oral infection. The cluster of acute appendicitis can be controlled and prevented by interventions targeting risk factors and the route of transmission without knowing the pathogen. The study may also be used for prevention of sporadic patients and be generalized in other populations. Future work will focus on finding new clusters to generalize the study and identify precipitating infectious agents.

## Data Availability Statement

The original contributions presented in the study are included in the article/Supplementary Material, further inquiries can be directed to the corresponding authors.

## Ethics Statement

The studies involving human participants were reviewed and approved by Ethic Boards at Infectious Disease Hospital Affiliated to Nanchang University and Wuhan University School of Medicine. Written informed consent to participate in this study was provided by the participants' legal guardian/next of kin.

## Author Contributions

YitG conceptualized and designed the study, acquired and analyzed clinical data, performed statistics, drafted the initial manuscript, and approved the final manuscript as submitted. DY acquired and analyzed clinical data, drafted the initial manuscript, and approved the final manuscript as submitted. GY and ST conceptualized and designed the study, analyzed clinical data, drafted the initial manuscript, and approved the final manuscript as submitted. GL conceptualized and designed the study, drafted the initial manuscript, and approved the final manuscript as submitted. XC acquired clinical data, drafted the initial manuscript, and approved the final manuscript as submitted. YiG conceptualized and designed the study, acquired and analyzed clinical data, drafted the initial manuscript, and approved the final manuscript as submitted. All authors approved the final manuscript as submitted and agree to be accountable for all aspects of the work.

## Conflict of Interest

The authors declare that the research was conducted in the absence of any commercial or financial relationships that could be construed as a potential conflict of interest.

## Publisher's Note

All claims expressed in this article are solely those of the authors and do not necessarily represent those of their affiliated organizations, or those of the publisher, the editors and the reviewers. Any product that may be evaluated in this article, or claim that may be made by its manufacturer, is not guaranteed or endorsed by the publisher.
